# Reviewed article: Araújo LBS, Sousa RA, Aguiar BGA, Mendonça VJ, Araújo OD, Tajra FS. Spatial and temporal distribution of neglected tropical diseases: overlap analysis, Piauí, 2013-2022. Epidemiol Serv Saude. 2026:35;e20250373

**DOI:** 10.1590/S2237-96222026v35e20250373.b

**Published:** 2026-03-16

**Authors:** Cristina Pissetti

**Affiliations:** 1 Universidade Federal da Paraíba, João Pessoa, PB, Brazil

## Peer review B

## Questionnaire

Does the manuscript contain new and significant information to justify publication? Yes

Does the Abstract (Summary) clearly and accurately describe the content of the article? Yes

Is the problem significant and concisely stated? Yes

Are the methods described comprehensively? No

Are the interpretations and conclusions justified by the results? Yes

Is adequate reference made to other work in the field? Yes

## Manuscript Structure

Length of article is: Adequate

Number of tables is: Adequate

Number of figures is: Adequate

Please state any conflict(s) of interest that you have in relation to the review of this paper. None

Interest: Good

Quality: Good

Originality: Good

Overall: Good

Recommendation: Minor Revision

## Comments

Prezados autores,

Como pontos positivos do manuscrito, podemos considerar:

- A escolha de analisar a sobreposição de doenças tropicais negligenciadas no Piauí é pertinente e preenche lacunas significativas na literatura científica sobre distribuição espaço-temporal dessas doenças.

- A abordagem quantitativa e analítica é apropriada para o objetivo proposto e o uso de dados do SINAN é uma escolha robusta para estudos epidemiológicos.

- O artigo apresenta objetivos claros e bem definidos, como a análise da tendência temporal e a caracterização sociodemográfica e espacial dos agravos.

Por outro lado, sentimos necessidade de melhores esclarecimentos acerca de alguns pontos, a saber:

- Explicar melhor como foram realizados os cálculos de incidência e prevalência.

Para o cálculo da incidência, não há uma descrição clara de como foram identificados e tratados os casos duplicados ou inconsistências no banco de dados, podendo impactar na precisão. O que foi considerado caso novo, visto que a coleta de dados foi em um único momento?

Para o cálculo da prevalência, não está claro se foram incluídos apenas os casos ainda ativos no período ou todos os registrados no sistema.

É importante discutir as limitações do estudo, como a possibilidade de vieses nos cálculos devido à subnotificação e duplicação de casos nos dados do SINAN. Houve algum ajuste para a subnotificação?

Na página 5, linha 46, sugere-se:

Posteriormente, para avaliar a sobreposição, os casos das doenças foram recodificados e unificados para visualização no espaço-tempo: se há pelo menos um caso, o código é igual a 1;se há 2 casos, 2 e assim sucessivamente, com um município podendo variar de 0 (nenhum caso dos agravos) até 6 (presença dos 6 agravos).

